# Computational Insights into Binding of Bisphosphates to Farnesyl Pyrophosphate Synthase

**DOI:** 10.2174/092986711794088335

**Published:** 2011-01

**Authors:** K Ohno, K Mori, M Orita, M Takeuchi

**Affiliations:** Drug Discovery Research, Astellas Pharma Inc., 21 Miyukigaoka, Tsukuba, Ibaraki 305-8585, Japan

**Keywords:** Bisphosphonate, farnesyl pyrophosphate synthase, bone resorption, osteoclast, protein-ligand binding, drug design.

## Abstract

Bisphosphonates (BPs) are the most widely used and effective treatment for osteoporosis and Paget's disease. Non-nitrogen containing BPs (non-N-BPs), namely etidronate, clodronate, tiludronate, as well as nitrogen-containing BPs (N-BPs), namely pamidronate, alendronate, ibandronate, risedronate, zoledronate and minodronate have been launched on the market to date. N-BPs act by inhibiting the enzyme farnesyl pyrophosphate synthase (FPPS), and several crystal structures of complexes between FPPS and N-BPs have been revealed. Understanding the physical basis of the binding between protein and small molecules is an important goal in both medicinal chemistry and structural biology. In this review, we analyze in detail the energetic basis of molecular recognition between FPPS and N-BPs. First, we summarize the interactions between ligands and proteins observed in N-BPs-FPPS complexes in the Protein Data Bank (PDB). Second, we present an interaction energy analysis on the basis of full quantum mechanical calculation of FPPS and N-BP complexes using the fragment molecular orbital (FMO) method. The FMO result revealed that not only hydrogen bond and electrostatic interaction but also CH-O and π-π interaction with FPPS are important for N-BP’s potency. Third, we describe a binding site analysis of FPPS on the basis of the inhomogeneous solvation theory which, by clustering the results from an explicit solvent molecular dynamics simulation (MD), is capable of describing the entropic and enthalpic contributions to the free energies of individual hydration sites. Finally, we also discuss the structure-activity relationship (SAR) of the series of minodronate derivatives.

## 1 INTRODUCTION

Minodronate (minodronic acid, YM-529, Ono-5920, YH-529), a third-generation Bisphosphonate (BP) originally developed at Astellas Pharma (formerly Yamanouchi), was approved and launched in 2009 in Japan for the oral treatment of osteoporosis. Minodronic acid, which was synthesized by a team of medicinal chemists lead by one of the authors (M. Takeuchi), has been shown to have 100- to 1000-fold higher bone resorption activity than pamidronate [1].

**Fig. (1):**
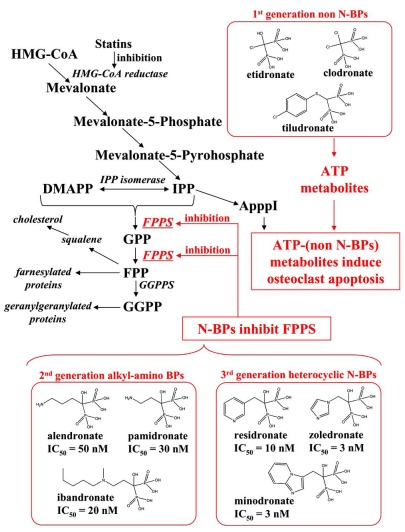
Schematic diagram of the mevalonate pathway and the chemical structures of bisphosphonate drugs. The formation of intracellular metabolites of non-N-BPs in osteoclasts induce osteoclast apoptosis, while N-BPs, including alkyl-amino BPs and heterocyclic N-BPs inhibit FPPS, thereby preventing the synthesis of FPP and GPP, which are required for protein prenylation. N-BPs also can induce formation of an ATP analog (ApppI) as a consequence of inhibition of the mevalonate pathway. The IC_50_ data was obtained from reference [16].

BPs are the most widely used and effective treatments for osteoporosis and Paget's disease [2, 3]. In addition to minodronate, etidronate (Proctor & Gamble, 1977), clodronate (Bayer, 1986), pamidronate (Gador/Novartis, 1987), alendronate (Abiogen/Merck, 1993), tiludronate (Sanofi-Aventis, 1995), ibandronate (Roche, 1996), risedronate (Sanofi-Aventis, 1998), and zoledronate (Novartis, 2000) have been launched on the market. Their chemical structures are shown in Fig. (**1**). Chemically, BPs are characterized by two phosphonate groups linked to a central carbon atom, forming a P-C-P structure [2,3]. They can be divided according to differences in the side chain into two groups, non-nitrogen containing BPs (non-N-BPs) and nitrogen-containing BPs (N-BPs), which are further classified into two additional groups, alkyl-amino BPs and heterocyclic N-BPs [4,5] (Fig. **1**). BPs inhibit osteoclast formation *in vitro*, disrupt the osteoclast cytoskeleton and ruffled border, and can cause osteoclast apoptosis [6]. The anti-resorptive potency of BPs has long been known to be influenced by the chemical structure of the side chain attached to the central carbon of the P-C-P backbone. It is thought that the mechanism of intracellular action differs between non-N-BPs and N-BPs. Non-N-BPs are metabolized in the osteoclast cytosol to non-hydrolyzable cytotoxic adenosine triphosphate (ATP) analogues that induce osteoclast apoptosis, while the more potent N-BPs inhibit farnesyl pyrophosphate synthase (FPPS), a key enzyme in the mevalonate pathway (Fig. **1**) [7-2]. FPPS (EC 2. 5. 1. 10) is a homodimeric enzyme of 41-kD subunits which catalyzes the sequential condensation of isopentenyl pyrophosphate (IPP), first with dimethylally pyrophosphate (DMAPP) and then with the resultant geranyl pyrophosphate (GPP) to produce the C_15_ farnesyl pyrophosphate (FPP). Inhibition of FPPS in osteoclasts prevents the biosynthesis of isoprenoid lipids that are required for the prenylation of small GTPase signaling proteins necessary for osteoclast function. Several crystal structures of human FPPS complexed with N-BPs have been reported (Table **1**). Similarly, N-BPs have been shown to inhibit farnesyl pyrophosphate/geranyl pyrophosphate synthase activity (GGPPS). Some N-BPs can inhibit GGPPS as well as FPPS [13].

The potency of a wide range of BPs to FPPS has been investigated [14]. To date, many researchers have analyzed the forces driving the formation of complexes between FPPS and N-BPs using experimental approaches. Yin *et al.* used isothermal titration calorimetry (ITC) to reveal the thermodynamic properties of binding between *T. burucei* FPPS and N-BPs, and showed that the binding of risedronate is enthalpy driven, whereas that of zoledronate is entropy driven [15]. Kavanagh *et al*. also applied the ITC method to reveal the thermodynamic properties of binding between human FPPS and N-BPs. In contrast to Yin *et al.* [15], they indicated that the binding of both risedronate and zoledronate are entropy driven [16]. The force driving the binding of FPPS and N-BPs thus remains unclear. In general, enthalpy-driven binding has several advantages over entropy-driven binding with regard to solubility and selectivity, and a deep understanding of the driving force would therefore facilitate the design of better compounds.

This review proceeds as follows. In the first section, we summarize the interaction between currently on-market bisphosphates and FPPS in Protein Data Bank (PDB). In the second section, we conduct an interaction energy analysis using the *ab initio* fragment molecular (FMO) method. In the third section, we analyze the hydration of binding site in FPPS on the basis of inhomogeneous solvation theory which, by clustering the results from an explicit solvent molecular dynamics (MD), is capable of describing the entropic and enthalpic contributions to the free energies of individual hydration sites. Finally, we discuss the structure-activity relationship (SAR) of a series of minodronate derivatives.

## 2 STRUCTURAL BASIS FOR THE RECOGNITION OF N-BPs BY FPPS

FPPS, one of the key regulatory enzymes in the mevalonate pathway and also the major target enzyme of N-BPs, is a single family which was first characterized by two aspartic-rich motifs with the consensus DDXXD sequence (X indicates any residues), which is highly conserved amongst isoprenyl synthases [17,18]. For catalytic activity, the presence of magnesium ions is required and Cornforth *et al.* have revealed that the reaction of FPPS occurs *via* a three-metal ion catalytic mechanism [17] (Fig. (**2a**)). The DDXXD motifs are important for ensuring the proper arrangement of three magnesium ions in the active site of FPPS [18].

Understanding the recognition of protein interaction sites is a critical step toward understanding the function of the protein, identification of functionally relevant amino acid residues, comparison and profiling of the ligands, and the structure-based drug design process. Recent technological innovations such as high-throughput crystallization and high performance synchrotron beam lines have enhanced the value of the technique further [19].

Crystallographic studies of human FPPSs of apo- and complex-forms with N-BPs (Table **1**) [16,20,21] revealed that (1) human FPPS is a homodimeric enzyme of 41-kDa subunits, (2) FPPSs have two substrate pockets, a DMAPP/GPP-binding site and an IPP-binding site (Fig. (**2b**)), (3) N-BPs bind to the DMAPP/GPP-binding pocket (Fig. (**2b**)), thereby acting as competitive inhibitors of this binding site as carbocation transition state analogs, (4) N-BPs’ binding causes a conformational change of FPPS which induces the closing of the active-site cavity by a rigid body motion of the last 130 C-terminal amino acids residues of the enzyme (Fig. (**2b**)), and (5) the binding of IPP causes fully closed conformation and stabilization of the FPPS-N-BP complexes.

**Fig. (3):**
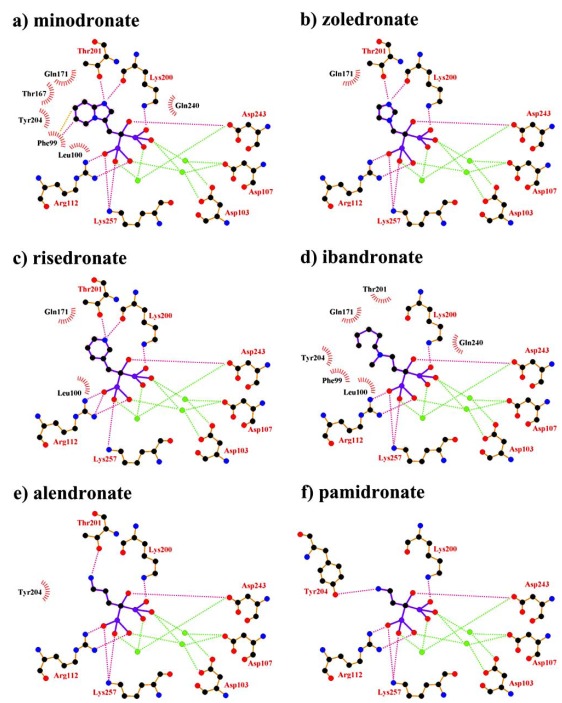
The interaction in N-BPs-FPPS complexes. **a**) LigPlot interactions in minodronate-FPPS complex (PDB id: 3B7L). **b**) LigPlot interactions in zoledronate-FPPS complex (PDB id: 2F8C). **c**) LigPlot interactions in risedronate-FPPS complex (PDB id: 1YV5). **d**) LigPlot interactions in ibandronate-FPPS complex (PDB id: 1F94). **e**) LigPlot interactions in alendronate-FPPS complex (PDB id: 1F92). **f**) LigPlot interactions in pamidronate-FPPS complex (PDB id: 1F89). Green circles represent metal ions, such as Mg^2+^, Zn^2+^, or Mn^2+^ ion. Dashed red lines shows the hydrogen bonding or polar interactions between N-BPs and FPPS and dashed green lines shows the interactions of the metal ions with N-BPs or FPPS. Dashed orange line show π-π interaction.

Fig. (**3**) shows schematic representations summarizing the binding of the N-BPs minodronate, zoledronate, risedronate, ibandronate, alendronate and pamidronate to FPPS. Images were created using the program LigPlot^+^ v.1.0, which generates schematic 2-D representations of protein-ligand complexes from the PDB file input [22]. The IDs of the PDB files used in the LigPlot calculation are 3B7L (minodronate), 2F8C (zoledronate), 1YV5 (risedronate), 2F94 (ibandronate), 2F92 (alendronate) and 2F89 (pamidronate), which are summarised in Table **1**. As shown in Fig. (**3**), all N-BPs have common P-C-P structures and their interactions are the same, wherein the bisphosphonate moiety of the N-BP binds to a trinuclear Mg^2+^ cluster (Fig. (**2a**)), and all three magnesium ions are octahedral coordination and linked with the carboxylate groups of the side chains of Asp103 and Asp107 in the first DDXXD motif and Asp243 in the second DDXXD motif, and water molecules. Two of three magnesium ions form rather symmetrical six-membered rings, with the bisphosphonate acting as a bidentate chelate, while one magnesium ion forms an additional single chelate. There are also direct salt bridge interactions of conserved residues Arg112, Lys200 and Lys257 of FPPS with the bisphosphonate moiety in all N-BPs. Because Lys257 is the residues in the HI loop (Fig. (**2b**)), the interaction between Lys257 and BPs is thought to contribute to stabilizing the closed conformation of FPPS. It is reported that the OH group on the carbon atom in the P-C-P structure in N-BPs, together with the two phosphate groups in this structure, is responsible for the N-BPs’ high affinity to bone mineral [23]. However, the OH group also contributes to FPPS binding *via *the polar interaction with Asp243 (Fig. **3**).

On the basis of the differences in the side chain, N-BPs can be classified into two groups [4, 5], the heterocyclic N-BPs, which include minodronate, zoledronate, and risedronate; and the alkyl-amino BPs, which include ibandronate, alendronate, and pamidronate (Fig. **1**). Using solid-state NMR, Mao *et al.* showed that N-BPs bind to FPPS *via* their protonated side chain [24]. Their results are supported by not only the crystallographic studies [1,16,20,21] but also our analysis using FMO calculation, which is described in the next section (direct interaction of Thr201). In all three heterocyclic N-BPs, minodronate, zoledronate and risedronate, the protonated heterocyclic nitrogen undergoes hydrogen bonding to both the hydroxyl group of Thr201 and the backbone carbonyl oxygen of Lys200 (Fig. (**3a**), (**3b**) and (**3c**)). The amino groups of alendronate and pamidronate, which belong to the alkyl-amino BPs, also interact with the hydroxyl groups of Thr201 and Tyr204, respectively (Fig. (**3d**) and (**3e**)). However, such bifurcated hydrogen bonding is only formed in heterocyclic N-BPs. These strong interactions of the protonated heterocyclic rings are thought to be one of the reasons why heterocyclic N-BPs have higher binding affinities for FPPS and greater inhibitory potency against the biochemical effect than alkyl-amino BPs. Fig. (**3**) also shows the hydrophobic contacts between FPPS and N-BPs. As shown in this figure, minodronate, which has a bulky bicyclic ring, and ibandronate, which has a long hydrophobic side chain, have a large number of hydrophobic contacts (Fig. (**3a**) and (**3d**)) compared with the other N-BPs. Moreover, these two N-BPs show tighter binding affinities than others in their respective classification (alkyl-amino BPs: ibandronate > alendronate, pamidronate; heterocyclic N-BPs: minodronate > zoledronate, risedronate). Minodronate is the only N-BP which has both bifurcated hydrogen bonding and a large number of hydrophobic contacts. These interactions are thought to make minodronate the most potent drug among BPs.

## 3 INTERACTION ANALYSIS BY FMO CALCULATION OF FPPS WITH N-BPs

We tried to understand the physical basis of the interaction between FPPS/N-BPs using *in silico* technology. There are two major *in silico* approaches: molecular mechanics (MM) and quantum mechanics (QM). The MM approach is very fast, but is limited by its inability to describe noncanonical interaction such as CH-π, CH-O hydrogen bond, and π-π correctly. In contrast, the QM approach overcomes the above issue, but its computational time is too long for practical use. The QM/MM method is one of the most promising methods of avoiding this problem [25]. However, QM/MM has its own problem, namely the boundary between the QM and MM parts, which causes an inevitable artificial error in energy. 

The fragment molecular orbital (FMO) method, originally developed by Kitaura, enables the analysis of a whole protein-ligand complex by quantum mechanical calculation without any of the issues described above.[26] The FMO method has been applied to many biomolecular systems to describe protein-ligand interaction in detail.[27,28] Here, to analyze the contribution from each amino acid residue to the interaction, we employed the FMO method as implemented in GAMESS to calculate the interaction energy of bisphosphonate compounds for FPPS. In the FMO method, a protein is divided into fragments based on the amino acid unit (Fig. (**S1a**)). It should be noted that the i-th fragment in FMO does not completely correspond to the i-th amino acid residue. The i-th fragment includes the carbonyl group of the (i-1)-th amino acid backbone but does not include that of the i-th. For example, the Thr201 fragment contains the Lys200 backbone carbonyl but does not contain carbonyl of Thr201. The contribution of the P-C-P part to total energy is negligible, because it is common in all N-BPs. Therefore, in the following paragraphs in this section, we focus on the interaction of N-BPs’ side-chain with these amino acids.

For accurate comparison of interaction energy, we used coordinates created by superposing the PDB structures over 3B7L and minimizing the proximate residues within 4.5 A from the ligand, which was replaced with minodronate. All water molecules were retained except for the molecule (residue number 1060 in 3B7L) in the ibandronate model, due to the molecule’s overlap with the heavy atoms of ibandronate. Mg910 in 3B7L seems to be a miss-assignment in X-ray modeling, because (1) a water molecule occupies this site in the other FPPS-ligand complexes, (2) no ligand for the Mg exists, and (3) no statement on metal coordination of Mg910 is written in the PDB data file. Therefore, we replaced Mg910 with a water molecule for FMO calculations. The coordinates of missing atoms and side chains were assigned by Maestro. Protonate3D in MOE was then used to assign the coordinate of hydrogen atoms of residues with a titratable group and polar hydrogen atoms. According to a previous study that demonstrated that N-BPs have a protonated side chain [24], the protonated form was used in FMO calculation. N-BPs was divided into two fragments (Fig. (**S1b**)), and FPPS was fragmented into one fragment per residue in the FMO standard fashion (Fig. (**S1a**)). All three Mg ions in the binding site were united into the P-C-P fragment of N-BP. Single point FMO2-MP2/6-31G calculations of all FPPS-N-BP complexes were carried out. Pair interaction energy decomposition analysis (PIEDA)[29] was used for analysis of detailed comparisons of interaction energy due to the difference in N-BP. FMO calculations were done using GAMESS version January 12, 2009, R3 for 64 bit Linux with Intel compilers [30].

First, we overviewed how the difference in N-BPs affected the interaction energy between FPPS and N-BPs. Fig. (**4**) shows fragment pair interaction energy difference (∆PIE) decomposed into each residue, taking minodronate as a reference. Having found several common regions which were affected by the difference in the side chain of N-BPs, we investigated in detail precisely which residue contributes to the interaction energy by calculating variance of PIE of each residue. Variance is a measure of how far vlaues are distributed from the mean. Therefore, the variance of PIE values with different N-BPs indicates the residue’s sensitivity to the difference in N-BPs. Fig. (**5a**) shows the variance of PIE of each residue. There were 14 residues mainly affected by the difference: Arg60, Phe99, Leu100, Asp103, Asp107, Arg112, Glu168, Gln171, Lys200, Thr201, Gln240, Asp243, Asp244, and Lys257. Interestingly, some of the residues with direct contacts as drawn in Fig. (**3**) were not among these 14 residues, indicating that not all of the residues near N-BP are important for binding or that current N-BPs cannot make use of all the residues. Surprisingly, the PIE of Asp103, Asp107, Arg112, Asp243, and Lys257 were correlated with potency (Fig. (**5b**)) although these residues have direct contact with only the common P-C-P part, indicating that the side-chain replacements of N-BPs have both direct and indirect effects on the interaction energy. We further investigated which residues vary and correlate with the potency of N-BPs by calculating covariance values between PIE and pIC50 (Fig. (**5b**)). Covariance is a measure of how much two variables vary together. Covariance differs from correlation in that covariance takes into consideration the magnitudes of two variables, whereas correlation normalizes the magnitudes. Given that, in the present study, we were interested only in those residues with PIE values varing largely in correlation with the potency, we adopted covariance over correlation as the preferred parameter to determine which residues have an impact on potency. The covariance between PIE and pIC50 of N-BPs clearly indicates that six residues tend to increase PIE as pIC50 becomes larger, namely Arg60, Leu100 (plus carbonyl oxygen atom of Phe99), Glu168, Lys200, Thr201, and Lys257. In addition, the covariance reveals that obtaining a smaller PIE with the other eight residues described above increases the potency of N-BPs. In the following paragraphs, we show the decomposition of these interactions and discuss them in detail.

### Direct Effect

In the 14 residues with large variance in PIE, Phe99, Leu100, Gln171, Lys200, and Thr201 have direct contact with the N-BP side chain. Moreover, four of the five have a correlation between the augmentation of their PIE and potency (Fig. (**5b**)). Moreover, it should be noted that, in FMO, the PIE of the hydrogen bond of the carbonyl oxygen atom of Lys200 is included in the PIE of Thr201. Because the only contact point of Lys200 with the N-BP sidechain is the backbone carbonyl oxygen atom, we focus here on describing the PIE of Phe99, Leu100, and Thr201. PIEDA decomposes PIE into an electrostatic (ES), exchange-repulsion (EX), charge transfer plus higher-order mixed term (CT+mix), and dispersion (DI). PIEDA can therefore describe any PIE in detail. Table **2** shows the result of PIEDA for Phe99, Leu100, and Thr201. 

Phe99 can be utilized well by minodronate and slightly by risedronate (Table **2**). Minodronate interacts with Phe99 *via* π-π interaction and *via* CH-O hydrogen bonding. The large nitrogen-containing electron-deficient imidazopyridine ring of minodronate allows to interact with the side chain of Phe99 *via* π-π interaction effectively. In addition to the fragment Phe99, minodronate shows remarkably large attractive energy with the fragment Leu100. It should be noted that minodronate transfers a much larger amount of charge (Table **2**). Looking into the complex structure (Fig. (**5c**)), we find that an aromatic hydrogen atom of the imidazopyrizine ring forms a CH-O bond with the backbone carbonyl group of Phe99, whose energy is integrated into the PIE of the fragment Leu100 in FMO. The finding of large ES and CT+mix terms also supports the notion that this interaction is achieved *via* hydrogen bonding. This results in the large attractive energy of minodronate for the fragment Leu100, because the sidechain of Leu100 interacts equally with the common methylene group of all the N-BPs. Therefore, Phe99 plays an important role in stabilizing N-BP binding, particularly for N-BPs with a large sidechain, like minodronate.

Hydrogen bonds with oxygen atoms of a backbone of Lys200 and sidechain of Thr201 is critical for the potency of N-BPs. In FMO, the interaction energy of the two hydrogen bonds is integrated into the PIE of the fragment Thr201. Table **2** indicates that the PIE of the fragment Thr201 depends almost completely on the ES term. This is reasonable because all N-BPs have a cationic nitrogen atom proximate to the oxygen atoms of Lys200 and Thr201. Interestingly, heterocyclic N-BPs have approximate 2-fold greater interaction than the others. This is due to the bifurcated hydrogen bonds with Lys200 and Thr201, which are characteristic of heterocyclic N-BPs, as described in the previous section.

To summarize the direct effects (as also shown in Fig. (**5d**)), the following interactions are likely important for the potency or N-BP: (1) hydrogen bonding with Thr201 and/or Lys200, with bifurcated hydrogen bonds likely to have greater potency than single bonds, (2) CH-O hydrogen bonding with the backbone carbonyl oxygen atom of Phe99, and (3) π-π interaction with the sidechain of Phe99. Among N-BPs, only minodronate satisfies all three conditions, which is likely one reason for its higher potency than the others.

### Indirect Effect

Fig. (**5b**) indicates that the potency of N-BPs correlates positively or negatively with the PIE of Arg60, Asp103, Asp107, Glu168, Lys200, Asp243, and Lys257. This is surprising because all have direct contact with the P-C-P portion of the N-BP and Mg ions, which are common in all N-BPs. In the FMO used in this study, their coordinates are completely identical. The seven residues have one unit of positive or negative charge on their sidechain. We therefore speculated that the difference in PIE might be caused by changes in the ES term of PIE due to (1) change of atomic charge on the P-C-P by sidechain replacement and (2) positive charge delocalization on the sidechain of N-BP. To investigate these possibilities, we performed PIEDA of the PIE of these residues at first (data not shown). The results indicated that PIE was dependent on the ES term. We then investigated which part of N-BPs affects the difference (Table **S1**). Results showed that ∆PIE of Arg60, Glu168, Lys100, Asp243, and Lys257 depends on the sidechain, while those of Asp103 and Asp107 depend on both the P-C-P and sidechain. Furthermore, we looked into the amount of charge of N-BPs transferred from the surrounding residues (Table **S2**). The results do not show a clear relationship with ∆PIE. It should be noted, however, that the basis set used in this study (6-31G) may underestimate the charge transfer associated with anions due to the lack of a diffuse function. Results may be improved by using a more extensive basis set, such as 6-31+G or 6-31+G*. Taking all results into account, the influence of the change in atomic charge due to charge transfer cannot explain the ∆PIE. We then investigated the delocalization of the positive charge on the sidechain of N-BP (Fig. **S2**). As expected, minodronate delocalizes its positive charge on the sidechain so that the positive charge reaches the tip of the heterocyclic ring, which decrease the electrostatic expulsion and attraction by the positively charged and negatively charged residues in the proximate P-C-P part of the N-BP, respectively. This delocalization leads to an increase in the electrostatic attraction energy between N-BP and Glu168, because Glu168 is located on the inverse side of the P-C-P binding site.

In summary, sidechain replacement of N-BP affects the electrostatic interaction with surrounding residues mainly due to the position of the positive charge on the sidechain. FMO calculation suggests that the delocalization tends to (1) stabilize interaction between N-BP and positively charged residues and (2) decrease electrostatic attractive energy by acidic residues, and (3) stabilize the interaction between N-BP and Glu168. Because the residues involved in the former two are located in the P-C-P binding site, the effect of (1) and (2) is trade-off relationship to ensure little impact on the increase in binding energy. FMO calculation suggests that Glul68 might be another contributor to the potency of N-BPs. However, Glul68 is exposed to solvent, and the electrostatical interaction might therefore be shielded.

## 4 HYDRATION IN THE POCKET OF FPPS

MD simulation is widely used to investigate the conformational change of proteins. For example, Sigman *et al*. conducted MD simulation for *T. cruzi* FPPS to reveal the conformational change induced by Mg^2+^ binding [31]. Recently, MD simulation of water molecules in active sites has gained considerable attention in the drug discovery field. When a ligand binds to a protein, the water in the active site is driven from the active site into the bulk fluid. This expulsion of the active-site solvent affects the binding free energy of the complex both enthalpically and entropically. When energetically or entropically unfavourable water molecules are expelled, the contribution of this expulsion to the binding free energy is favourable. Recently, Friesner’s group developed a novel, computationally efficient method to analysis environments for solvating water in the active site pockets (WaterMap method) [32]. The WaterMap method can provide deep insights into molecular recognition at low computational cost. To date, several applications of this method have been developed [33,34]. These have shown that the active sites of COX-2 and Factor Xa provide very diverse environments for solvating water in the active site. Furthermore, the calculated relative difference in binding free energy is well consistent with the experimental one.

To built an input file for the WaterMap method, each three-dimensional coordinate of the FPPS/BP complex was obtained from Protein Data Bank (Table **1**). The addition of hydrogen atoms was conducted using MOE 2006.08 [35] and the energy minimization calculation was carried out using Maestro 8.5 ed. (Schrodinger) [36]. The WaterMap method has been described in detail elsewhere [33]. MD simulations were conducted using the Desmond molecular dynamics engine [37]. The OPLS2005 force field was used [38,39]. The energy minimized structure was used as a initial coordinate. Atoms of FPPS were truncated beyond 15 Å of the ligand. The ligand was not included in the system. The resulting protein construct was solvated in a TIP4P water box extending at least 5 Å beyond the FPPS in all directions. 2ns MD simulation with positional restraints on the protein non-hydrogen atoms was performed following the default relaxation protocol, including successive stages of minimization and heating. Water molecules in the active site were clustered to form hydration sites. The enthalpy term was obtained from the average non-bonded energy of each hydration site. The excess entropy term was obtained by numerically integrating a local expansion of spatial and orientational correlation functions. [32,40,41] In the entropy calculation, only contributions from the first order term of the expansion were taken into account. Ligand binding energies were then estimated as the sum of hydration site (hs) free energies that are displaced by ligand atoms (lig) upon binding. The function for the G of binding is

(eq. 1) ΔGbind = ∑lig,hsΔGhs 1−r→lig−r→hsRCOΘRCO−r→lig−r→hs

where, *R_co_* is the cutoff distance for a ligand atom to begin displacing a hydration site, *G_hs_* is the computed free energy of transferring the water molecule in a given hydration site from the active site to the bulk fluid, and *Θ* is the Heaviside step function. The value of Rco was selected as 2.24. [32] 

The results for each hydration site are summarized in Table **S3**. There are large differences in the thermodynamic properties of hydration sites, which indicates that the active site of FPPS provides very diverse environments for solvating water. As stated by Friesner’s group and researchers at Schrodinger Inc., those of COX-2, Factor Xa and the PDZ domain also provide markedly varied environments for active site water molecules [32-34]. As shown in Table **S3**, the enthalpy contributions of hydration site 12 and 20 have positive values, which indicates that water molecules in these hydration sites are enthalpically unfavourable. Thus, the expulsion of the water molecule in these hydration sites makes an enthalpically favourable contribution to binding free energy. In contrast, the enthalpy contributions of hydration site 1, 2, 5, and 6 are under -7 kcal/mol, which indicates that water molecules in these hydration sites are enthalpically favourable. Thus, the expulsions of water molecules in these hydration sites makes an enthalpically unfavourable contribution to binding free energy. Although the enthalpic contributions of almost all hydration sites have negative values, those of FPPS are somewhat larger than those of Factor Xa [32]. On the other hand, the entropic contribution of each hydration site has a positive value. This means that the expulsion of water molecules has an entropically favourable effect on binding free energy.

Fig. (**6**) shows the three-dimensional hydration map. In Fig. (**6a**), hydration sites whose ∆G values were less than -3.0 kcal/mol (1, 5, 6, 7, 13, 14, and 18) are shown in green cubes. These hydration sites overlapped the P-C-P backbone structure. In Fig. (**6b**), the hydration sites whose ∆H have a positive value (12 and 20) are shown in red cubes. These hydration sites overlapped the imidazopyridine ring of minodronate. Details of the properties of individual sites are shown in supplemental material Table **S4**. In the case of hydration sites 12 and 20, there were large differences in thermodynamics properties among inhibitors. For example, in the case of hydration site 12, the ∆G values of minodronate and ibandronate were somewhat smaller than those of zoledronate, alendronate, and pamidronate, by 4 kcal/mol. In the case of hydration site 20, those of minodronate and ibandronate were also somewhat smaller than those of alendronate and pamidronate, by 4 kcal/mol. These differences arise from the fact that minodronate and ibandronate can expel the unstable water molecules in hydration sites 12 and 20 (Fig. (**6b**)) whereas alendronate and pamidronate cannot. In contrast, in the case of hydration site 6, ∆G is quite large, and there is no difference among BPs. These results indicate that although the expulsion of water molecules in hydration site 6 has a large effect on the binding free energy, all BPs can expel the water molecule in hydration site 6.

The purpose of this part of the study was to investigate the energetic basis of molecular recognition between FPPS and BPs using MD calculations of solvating water molecules. From the results of MD calculation, the environment for solvating water molecules was shown to be very diverse. Furthermore, we showed that minodronate and ibandronate can expel water molecules in these hydration sites (12 and 20), which usually cause a significant affinity increase. It is noteworthy that minodronate is the only bisphosphonate which has the following two merits: (1) exclusion of unstable water molecules in the active site pocket and (2) strong interaction with Lys200 and Thr201, as mentioned in the previous section. In fact, minodronate has the strongest activity *in vitro* and *in vivo* among launched BPs. 

## 5 RE-CONSIDERATION OF STRUCTURE-ACTIVITY RELATIONSHIP OF HETEROCYCLIC N-BPs

**Fig. (7):**
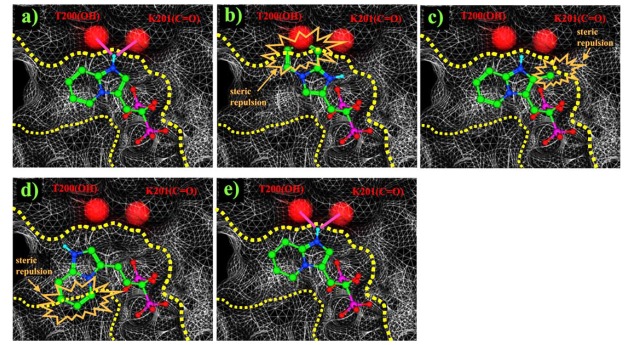
Molecular modeling studies of compounds 1-4. **a**) Experimental binding mode of minodronate observed in the crystal structure (PDB id: 3B7L). **b**) Examination of the binding of compound 1 using molecular modeling. **c**) Examination of the binding of compound 2 using molecular modeling. **d**) Examination of the binding of compound 3 using molecular modeling. **e**) Examination of the binding of compound 4 using molecular modeling. Dashed yellow lines indicate the surface of the binding site of the protein. Red circles are the hydroxyl group of Thr201 and the backbone carbonyl oxygen of Lys200. Pink lines indicate the hydrogen bonding favourable to the tight binding to FPPS. Steric repulsion, which is unfavourable for binding, is also shown as orange lines.

In early research into BPs conducted since the late 1960s by many pharmaceutical companies, the target of BPs was unknown and BPs were thought to act by modulating bone mineral dissolution [42]. The SAR of activity in inhibiting bone resorption was therefore the only information for medicinal chemists, and the structure-based drug design (SBDD) approach using the three dimensional structure of the target enzyme and SAR analysis using direct *in vitro* activity of enzyme inhibition could not be applied. Table **3** shows our previously published data on the structures of our heterocyclic series of minodronate derivatives [1,43], their clogP values of the side chain, and their experimental antiresorptive activities. Our early research found that the bulkiness and lipophilicity of the side chain of N-BPs were important determinants of their antiresoptive activity [43]. However, compound 1, having an equivalent clogP value, an index of lipophilicity as minodronate, and the compounds 2 and 3, having clogP values greater than that of minodronate, showed remarkably decreased potency, whereas compound 4, having clogP values below that of minodronate, showed almost the same level of activity as minodronate. It is difficult to interpret the SAR of these compounds using only their activity in inhibiting bone resorption. Now, however, use of the three-dimensional structure of FPPS, the target of N-BPs, has clarified this issue. Moreover, Dunford *et al*. reported a high correlation between the order of inhibition of human FPPS *in vitro* and antiresorptive activities *in vivo* [14], and Rogers also reported the correlation coefficient (r) as 0.95 [44]. In section 2 of this review, we noted that the bifurcated hydrogen bonding of the protonated heterocyclic nitrogen to the hydroxyl group of Thr201 and the backbone carbonyl oxygen of Lys200 is important for the tight binding for FPPS (Fig. (**3a**), (**3b**) and (**3c**)). From the molecular modeling studies showing that compounds 1, 2 and 3 undergo steric repulsion with the binding pocket of FPPS, it is clear that compounds 1-3 cannot make such hydrogen bonds, while the protonated heterocyclic nitrogen of compound 4 can undergo bifurcated hydrogen bonding (Fig. **7**). As shown in this section, our re-consideration of the SAR of heterocyclic N-BPs in Table **3** is useful in facilitating the further optimization of N-BPs, and proves the importance of identifying the drug target.

## 6 CONCLUSION

Although BPs are a major class of drugs for the treatment of diseases characterized by excessive osteoclast-mediated bone resorption, such as post-menopausal and steroid-induced osteoporosis, the difficulty of isolating a large number of purified osteoclasts for biochemical studies and the diverse effects of BPs on osteoclasts have meant that the molecular mechanisms of action of these drugs have remained unclear for more than 40 years. As described in this review, BPs are classified into two groups, non-N-BPs and N-BPs. Several biological efforts have revealed that non-N-BPs are metabolically incorporated into non-hydrolysable ATP and induce osteoclast apoptosis, while the major mechanism by which N-BPs inhibit bone resorption is *via* the inhibition of FPPS enzyme Fig. (**1**). Moreover, structural biological efforts, including crystallographic studies, have clarified the interactions between FPPS and many N-BPs (Table **1**). Now, many crystal structures of FPPS complexed with N-BPs have been deposited in the PDB, making possible the computational simulation approaches about N-BPs described here, including our own research.

In this review, we first provided a detailed analysis of the molecular interactions between FPPS and six N-BPs, namely minodronate, zoledronate, risedronate, ibandronate, alendronate and pamidronate, in their crystal structure Fig. (**3**). These analyses confirmed the structure-activity relationships we previously obtained in our synthesis research (Table **3**).

The FMO method has indicated that the following interactions may be important for the potency of N-BPs: (1) hydrogen bonding with Thr201 and/or Lys200, where a bifurcated bond would increase potency over a single bond, (2) CH-O hydrogen bonding with the backbone carbonyl oxygen atom of Phe99, and (3) π-π interaction with the sidechain of Phe99. Moreover, the difference in the sidechain could affect the electrostatic interaction with the surrounding amino acids indirectly. In N-BPs, only minodronate satisfied these conditions, which is likely one reason why minodronate shows the highest potency against FPPS among all the N-BPs. 

The hydration of the binding site in FPPS has been investigated using the binding site analysis in FPPS on the basis of the inhomogeneous solvation theory which, by clustering the results from an explicit solvent MD, is capable of describing the entropic and enthalpic contributions to the free energies of individual hydration sites. This analysis showed that the strong activity of minodronate and ibandronate arises from the exclusion of unstable solvating water molecules. Although many methods of predicting affinity have been proposed, few methods take consideration of the entropy contribution of solvating water molecules. Hydration site analysis can provide deep insights into the binding between protein/ligand, although its prediction of relative binding free energies is not particularly accurate. Combinational use with QM or MM/GBSA may be desirable.

The use of three-dimensional structural information of a target protein in the process of drug development, which is also called SBDD, is a highly attractive strategy for pharmaceutical companies. The availability of new crystal structures of drug targets is therefore of broad general interest in the context of the rational development of potent inhibitors, and the identification of the drug target, as well as their structural determination, are thought to be crucial steps in recent rational drug research. Because many approaches to target profiling of small molecules are used in both academic and pharmaceutical research laboratories, it is clear that many targets of drugs in clinical use or development will be found. In this review, we show a detailed analysis of the energetic basis of molecular recognition between FPPS and N-BPs. It is expected that similar research on the analysis of interactions between BP drugs and FPPS will increase in the future, and facilitate further drug research.

## SUPPLEMENTARY MATERIAL

Supplementary material is available on the publishers Web site along with the published article.

## Figures and Tables

**Fig. (2):**
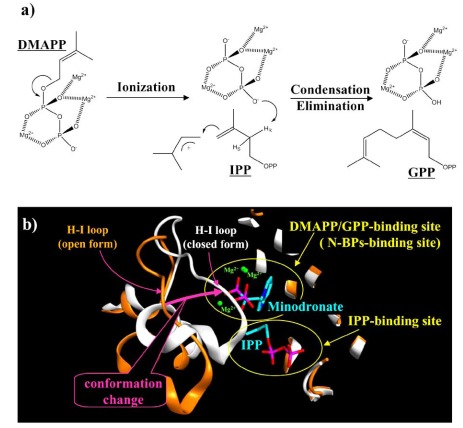
**a**) A schematic showing three-metal ion catalytic mechanism of the FPPS enzyme. **b**) three-dimensional view of DMAPP/GPPbinding site and IPP-binding site of our modelled complex of minodronate (blue), IPP (blue) and closed form of FPPS (white ribbon). This model was constructed using the crystal structures of minodronate-FPPS complex (PDB id: 3B7L) and zoledronate-IPP-FPPS complex (PDB id: 2F8Z) as templates. The orange ribbon shows the open form of unligated FPPS (PDB id: 2F7M). The pink arrow indicates the conformation change induced by the ligand binding.

**Fig. (4):**
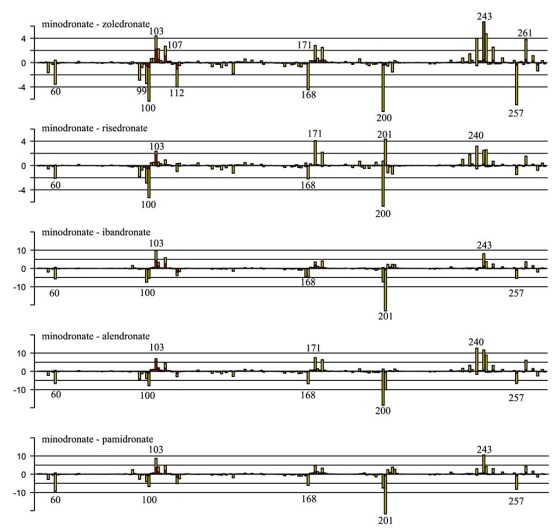
ΔPIE (in kcal/mol) of N-BP with FPPS. Yellow and red bars represent contribution of sidechain and P-C-P fragments, respectively. For clarity, ΔPIE values of N- and C-terminal residues are not shown, as no significant difference was observed.

**Fig. (5):**
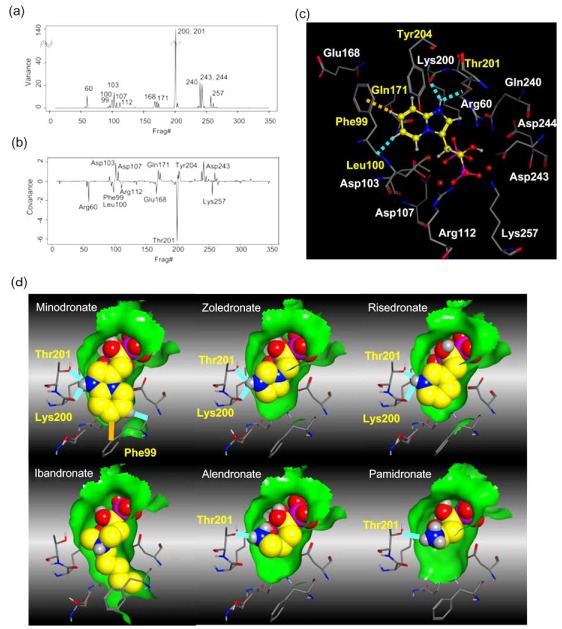
Residues sensitive to the difference in N-BP side chain. (**a**) Variance of PIE of each residue (unit is square of kcal/mol). (**b**) Covariance between PIE of each residue and pIC50 of N-BPs (unit is –log_10_(mol)*kcal/mol). (**c**) Molecular graphics of FPPS and minodronate. The residues with large variance in PIE are shown. The atoms are colored according to the element, and the carbon atoms of minodronate are colored yellow. The residues with direct contacts with the N-BP sidechain are labeled in yellow and others in white. Hydrogen bonds and π-π interactions are represented by sky-blue and orange dashed lines. (**d**) Summary of the direct effect achieved with sidechain replacement of NBPs. Hydrogen bonds and π-π interaction are represented by sky-blue and orange lines. The residues involved in the hydrogen bonding are labeled in yellow. Non-polar hydrogen atoms, hydrogen atoms involved in hydrogen bonding, and heavy atoms are also shown. The green surface represents the molecular surface of the binding pocket. For clarity, the molecular surface of the pocket in front of the N-BPs has been deleted.

**Fig. (6):**
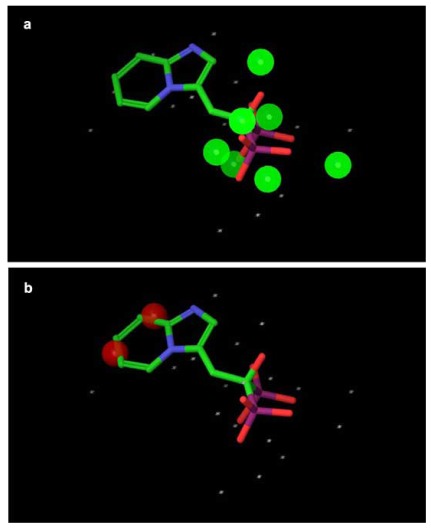
The hydration sites in the active site of FPPS. (**a**) Minodronate and hydration sites whose ΔG values were below -3.0 kcal/mol; (**b**) minodronate and hydration sites whose ΔH had a positive value.

**Table 1:** Summary of FPPS Structures

pdb code	resolution/Å	ligand	mutation	calculation	reference
3B7L	1.95	minodronate, Mg		Yes	[20]
2VF6	2.10	minodronate, Mg			[20]
2F8C	2.20	zoledronate , Mg		Yes	[21]
2F8Z	2.60	zoledronate, Mg, IPE			[21]
2F9K	2.06	zoledronate, Zn, PO4			[21]
1ZW5	2.30	zoledronate, Mg, IPR			[16]
1YV5	2.00	risedronate, Mg, PO4		Yes	[16]
1YQ7	2.20	risedronate, Mg, PO4			[20]
2QIS	1.80	risedronate, Mg	T210S		[20]
2F94	1.94	ibandronate, Zn, PO4		Yes	[21]
2F92	2.15	alendronate, Zn, PO4		Yes	[21]
2F89	2.60	pamidronate, Mn, PO4		Yes	[21]

IPE, IPR and PO4 mean 3-methylbut-3-enyl trihydrogen diphosphate, isopentyl pyrophosphate, and phosphate ion, respectively. In this report, 3B7L, 2F8C, 1YV5, 2F94, 2F92, and 2F89 were used for calculation.

**Table 2:** Decomposed Pair Interaction Energy (in kcal/mol) and Charge Transfer (in a.u.) between the Sidechain of N-BPs and FPPS

Fragmenta)	Inhibitor	Total	ESb)	EXb)	CT+mixb)	DIb)	DQc)
Phe99	minodronate	-2.8	-2.5	5.0	-1.6	-3.7	0.0047
	zoledronate	0.10	0.31	0.0010	-0.036	-0.17	0.00
	risedronate	-0.39	0.086	0.017	-0.090	-0.41	0.00
	ibandronate	4.4	-7.1	23	-3.0	-8.7	0.013
	alendronate	0.50	0.61	-0.0010	-0.010	-0.090	0.00
	pamidronate	0.77	0.85	-0.0010	-0.0090	-0.069	0.00
Leu100	minodronate	-11	-9.8	4.7	-1.7	-4.1	0.019
	zoledronate	-5.3	-4.4	0.76	-0.45	-1.2	0.0021
	risedronate	-6.2	-5.1	1.2	-0.57	-1.7	0.0023
	ibandronate	-6.0	-5.9	4.2	-1.2	-3.1	0.0069
	alendronate	-4.0	-3.3	1.2	-0.52	-1.3	0.0020
	pamidronate	-4.9	-4.3	1.1	-0.56	-1.2	0.0032
Thr201	minodronate	-45	-45	10	-3.9	-5.9	0.056
	zoledronate	-45	-43	6.4	-3.2	-4.7	0.042
	risedronate	-49	-58	21	-5.2	-6.3	0.067
	ibandronate	-22	-28	13	-2.9	-4.8	0.035
	alendronate	-35	-43	16	-5.5	-3.3	0.054
	pamidronate	-24	-22	0.078	-0.82	-0.81	0.0065

a The i-th fragment in FMO does not completely correspond to the i-th amino acid residue. The i-th fragment includes the carbonyl group of the (i-1)-th amino acid backbone but not that of the i-th.

b ES, EX, CT+mix, DI represent electrostatic, exchange-repulsion, charge transfer plus higher-order mixed terms, and dispersion contribution, respectively.

c ΔQ is amount of charge transferred from the residue to side chain of N-BP. Charges are derived from two-body Mulliken atomic charges calculated using the FMO2-MP2/6-31G calculation.

**Table 3:** Effects of Minodronate Derivatives in Antiresorptive Activity 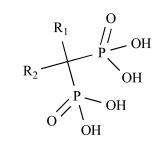

compound	structure of R_1_ group	structure of R_2_ group	clogP of R groupa)	antiresorptive activityb) (mg/kg, s.c.)
minodronate	OH	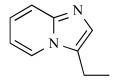	1.468	0.003
1	OH	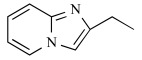	1.468	> 0.1
2	OH	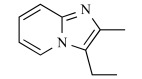	1.687	> 0.3
3	OH	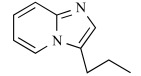	1.997	> 0.3
4	OH	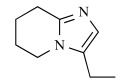	1.099	≤ 0.01

a The methylene and ethylene groups between aromatic rings and bisphosphonate groups were calculated as methyl and ethyl groups, respectively.

b Antiresorptive activities were evaluated using the parathyroid hormone (PTH)-induced hypercalcemia model (PIH model) [1].

## ABBREVIATIONS

ApppI: = triphosphoric acid 1-adenosin-5’-yl ester 3-(3-methylbut-3-enyl) ester
BPs: = Bisphosphonates
CT+mix: = Charge transfer plus higher-order mixed terms
DI: = Dispersion
DMAPP: = Dimethylally Pyrophosphate
ES: = Electrostatic
ESP: = Electrostatic potential
EX: = Exchange-repulsion
FMO: = Fragment Molecular Orbital
FPPS: = Farnesyl Pyrophosphate Synthase
GPP: = Geranyl Pyrophosphate
IPP: = Isopentenyl Pyrophosphate
ITC: = Isothermal titration calorimetry
N-BPs: = Nitrogen-containing BPs
PIEDA: = Pair interaction energy decomposition analysis
