# Supplementary material for: Computational Insights into Binding of Bisphosphates to Farnesyl Pyrophosphate Synthase
Source: Curr Med Chem. 2011 Jan;18(2):220–33. doi: 10.2174/092986711794088335 (PMC3343387; doi:10.2174/092986711794088335)
Supplement: Supplementary file 1 [file CMC-18-220_SD1.pdf]

SUPPLEMENTARY MATERIAL

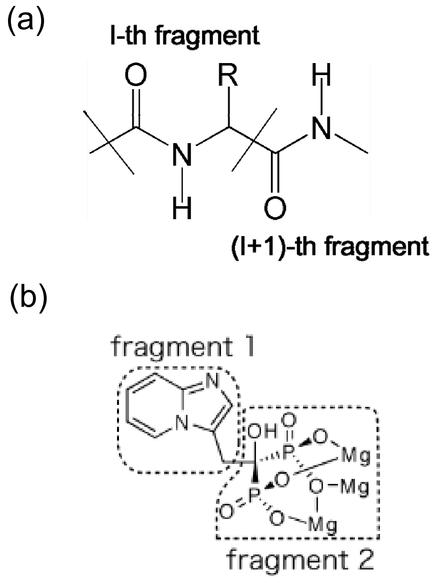

Fig. (S1). Fragmentation in FMO. (**a**) Fragmentation of protein. (**b**) Fragmentation of N-BP.

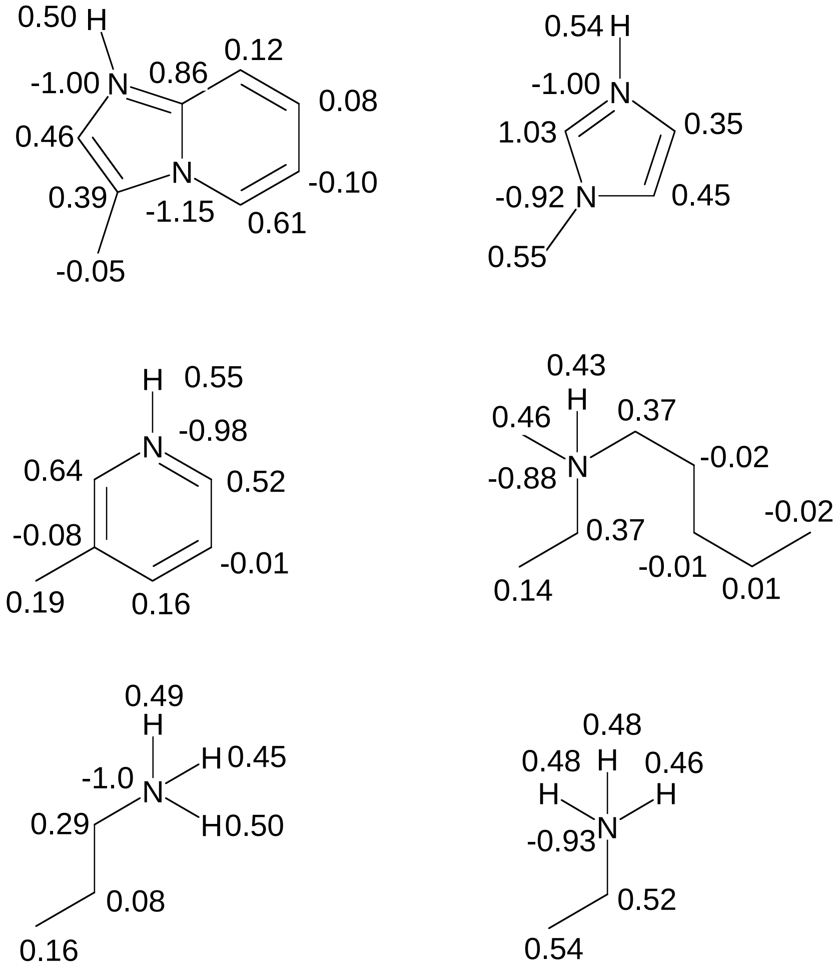

Fig. (S2). Atomic charge on the sidechain of N-BPs. Charges are derived from two-body Mulliken atomic charges calculated by FMO2-MP2/6-31G calculation. The atomic charges of hydrogen atoms except polar hydrogen atoms are united into the carbon atom.

Table S1. Pair Interaction Energy Difference (in kcal/mol) of N-BPs from Minodronate

Residue	Inhibitor	ΔPIE(PCP)	ΔPIE(SC)	ΔPIE(PCP+SC)
Arg60	zoledronate	-0.419	3.96	3.541
	risedronate	-0.087	2.171	2.084
	ibandronate	-0.585	6.274	5.689
	alendronate	-0.504	7.183	6.679
	pamidronate	-0.627	9.983	9.356
Asp103	zoledronate	-2.202	-2.21	-4.412
	risedronate	-1.747	-0.611	-2.358
	ibandronate	-4.278	-5.558	-9.836
	alendronate	-5.499	-1.346	-6.845
	pamidronate	-3.474	-5.116	-8.59
Asp107	zoledronate	-1.111	-1.612	-2.723
	risedronate	-0.198	-0.73	-0.928
	ibandronate	-2.468	-3.347	-5.815
	alendronate	-1.719	-2.605	-4.324
	pamidronate	-1.961	-2.929	-4.89
Lys200	zoledronate	0.415	7.605	8.02
	risedronate	-0.736	7.409	6.673
	ibandronate	-0.469	7.823	7.354
	alendronate	-1.149	19.715	18.566
	pamidronate	0.033	7.539	7.572
Asp243	zoledronate	-0.475	-8.614	-9.089
	risedronate	-0.219	-2.283	-2.502
	ibandronate	-1.273	-6.765	-8.038
	alendronate	-0.69	-10.922	-11.612
	pamidronate	-0.788	-9.678	-10.466
Lys257	zoledronate	0.405	6.51	6.915
	risedronate	0.355	1.123	1.478
	ibandronate	0.921	4.488	5.409
	alendronate	0.939	5.739	6.678
	pamidronate	0.901	7.41	8.311

SC stands for side chain.

Table S2. Charge Transfer (in a.u.) to N-BPs from FPPS

Inhibitor	ΔQ(PCP+Mg)	ΔQ(SC)	ΔQ
minodronate	2.4927	0.0923	2.585
zoledronate	2.4501	0.1329	2.583
risedronate	2.4678	0.1051	2.5729
ibandronate	2.5147	0.2024	2.7171
alendronate	2.4668	0.1717	2.6385
pamidronate	2.4725	0.1241	2.5966

SC stands for side chain.

Table S3. Calculated Thermodynamic and Local Water Structure Data for Each of the 20 Hydration Sites (in kcal/mol)

# site	ΔH	-TΔS	ΔG
1	-8.53	5.28	-3.25
2	-8.23	5.31	-2.92
3	-4.25	4.04	-0.21
4	-5.51	4.32	-1.19
5	-7.47	3.64	-3.83
6	-11.49	3.72	-7.77
7	-6.72	3.59	-3.13
8	-1.25	2.73	1.48
9	-1.56	2.62	1.06
10	-5.27	4.05	-1.22
11	-2.16	2.32	0.16
12	1.78	2.42	4.2
13	-6.18	2.65	-3.53
14	-5.91	2.58	-3.33
15	-4.73	2.38	-2.35
16	-1.74	2.88	1.14
17	-2.19	1.61	-0.58
18	-4.52	1.5	-3.02
19	-3.34	1.24	-2.1
20	2.92	1.13	4.05

ΔH is average energy of interaction of the water molecules with the rest of the system in a given hydration site. −TΔS is the excess entropic contribution to the free energy calculated from a truncated expansion of the excess entropy in terms of correlations in the single particle translational and rotational density. ΔG is the total free energy (ΔG = ΔH – TΔS).

Table S4. The Thermodynamics Properties for Individual Sites (in kcal/mol)

site	property	minodronate	zoledronate	risedronate	ibandronate	alendronate	pamidronate
1	-T Δ S	-5.3	-5.3	-5.2	-5.3	-5.3	-5.3
	Δ H	8.5	8.5	8.4	8.5	8.5	8.5
	Δ G	3.2	3.2	3.2	3.2	3.2	3.2
2	-T Δ S	0.0	0.0	0.0	0.0	0.0	0.0
	Δ H	0.0	0.0	0.0	0.0	0.0	0.0
	Δ G	0.0	0.0	0.0	0.0	0.0	0.0
3	-T Δ S	-0.6	-0.6	-0.8	-1.0	-0.5	-0.3
	Δ H	0.6	0.6	0.8	1.0	0.6	0.3
	Δ G	0.0	0.0	0.0	0.1	0.0	0.0
4	-T Δ S	-2.8	-2.6	-2.8	-2.3	-2.3	-2.2
	Δ H	3.5	3.3	3.6	3.0	2.9	2.8
	Δ G	0.8	0.7	0.8	0.6	0.6	0.6
5	-T Δ S	-3.0	-2.8	-2.7	-2.5	-2.5	-3.5
	Δ H	6.1	5.8	5.5	5.2	5.1	7.2
	Δ G	3.1	3.0	2.8	2.7	2.6	3.7

(Table S4). Contd…..

site	property	minodronate	zoledronate	risedronate	ibandronate	alendronate	pamidronate
6	-T Δ S	-3.7	-3.7	-3.7	-3.7	-3.7	-3.7
	Δ H	11.5	11.5	11.5	11.5	11.5	11.5
	Δ G	7.8	7.8	7.8	7.8	7.8	7.8
7	-T Δ S	-0.2	-0.3	0.0	0.0	0.0	-0.1
	Δ H	0.3	0.5	0.0	0.0	0.0	0.2
	Δ G	0.2	0.2	0.0	0.0	0.0	0.1
8	-T Δ S	-2.7	-2.7	-2.7	-2.7	-2.7	-2.7
	Δ H	1.2	1.2	1.2	1.2	1.2	1.2
	Δ G	-1.5	-1.5	-1.5	-1.5	-1.5	-1.5
9	-T Δ S	-2.2	-2.3	-1.6	-2.0	-2.3	-1.5
	Δ H	1.3	1.4	1.0	1.2	1.3	0.9
	Δ G	-0.9	-0.9	-0.7	-0.8	-0.9	-0.6
10	-T Δ S	0.0	0.0	0.0	-0.1	-0.2	0.0
	Δ H	0.0	0.0	0.0	0.2	0.2	0.0
	Δ G	0.0	0.0	0.0	0.0	0.1	0.0
11	-T Δ S	-2.3	-2.2	-2.1	-2.0	-1.9	-2.0
	Δ H	2.2	2.0	2.0	1.9	1.8	1.9
	Δ G	-0.2	-0.2	-0.2	-0.1	-0.1	-0.1
12	-T Δ S	-2.4	0.0	-0.8	-2.4	0.0	0.0
	Δ H	-1.8	0.0	-0.6	-1.8	0.0	0.0
	Δ G	-4.2	0.0	-1.5	-4.2	0.0	0.0
13	-T Δ S	-2.6	-2.6	-2.6	-2.6	-2.6	-2.6
	Δ H	6.2	6.2	6.2	6.2	6.2	6.2
	Δ G	3.5	3.5	3.5	3.5	3.5	3.5
14	-T Δ S	-0.6	-0.6	-0.7	-0.5	-0.5	-0.6
	Δ H	1.4	1.3	1.7	1.0	1.1	1.4
	Δ G	0.8	0.8	0.9	0.6	0.6	0.8
15	-T Δ S	-2.4	-2.4	-2.4	-2.4	-1.6	0.0
	Δ H	4.7	4.7	4.7	4.7	3.1	0.0
	Δ G	2.3	2.3	2.3	2.3	1.5	0.0
16	-T Δ S	-0.4	0.0	0.0	-2.9	0.0	0.0
	Δ H	0.3	0.0	0.0	1.7	0.0	0.0
	Δ G	-0.2	0.0	0.0	-1.1	0.0	0.0
17	-T Δ S	0.0	-0.1	0.0	0.0	0.0	0.0
	Δ H	0.0	0.1	0.0	0.1	0.0	0.0
	Δ G	0.0	0.0	0.0	0.0	0.0	0.0
18	-T Δ S	-1.5	-1.5	-1.5	-1.5	-1.5	-1.5
	Δ H	4.5	4.5	4.5	4.5	4.5	4.5
	Δ G	3.0	3.0	3.0	3.0	3.0	3.0
19	-T Δ S	-0.1	-0.2	-0.2	-0.2	-0.1	-0.1
	Δ H	0.4	0.5	0.4	0.5	0.3	0.4
	Δ G	0.2	0.3	0.3	0.3	0.2	0.2
20	-T Δ S	-1.1	-0.8	-1.1	-1.1	0.0	0.0
	Δ H	-2.9	-2.0	-2.9	-2.7	-0.1	0.0
	Δ G	-4.1	-2.8	-4.1	-3.8	-0.1	0.0
